# The acceptability of targeted mass treatment with primaquine for local elimination of vivax malaria in a northern Myanmar township: a mixed-methods study

**DOI:** 10.1186/s13071-021-05064-y

**Published:** 2021-10-24

**Authors:** Pyae Linn Aung, Myat Thu Soe, Than Naing Soe, Thit Lwin Oo, Poh Poh Aung, Aung Khin, Aung Thi, Suparat Phuanukoonnon, Kamolnetr Okanurak, Liwang Cui, Myat Phone Kyaw, Daniel M. Parker

**Affiliations:** 1 Myanmar Health Network Organization, Yangon, Myanmar; 2 Department of Public Health, Ministry of Health, NayPyiTaw, Myanmar; 3 Department of Social and Environmental Health, Faculty of Tropical Medicine, Mahidol University, Bangkok, Thailand; 4 Division of Infectious Diseases and International Medicine, Department of Internal Medicine, Morsani College of Medicine, University of South Florida, 3720 Spectrum Boulevard, Suite 304, Tampa, FL 33612 USA; 5 Department of Population Health and Disease Prevention, Department of Epidemiology, University of California, Irvine, CA USA

**Keywords:** Acceptability, Targeted mass treatment, Primaquine, Mixed-methods, Myanmar

## Abstract

**Background:**

Radical cure of the *Plasmodium vivax* latent liver stage is required to effectively manage vivax malaria. Targeted mass treatment with primaquine may be an effective mechanism for reducing reservoirs of the disease. Since community engagement and high coverage are essential for mass treatment programs, this study aimed to determine the acceptability of mass primaquine treatment in a targeted community in a northern Myanmar township.

**Methods:**

A cross-sectional mixed-methods study was deployed among household leaders in July 2019. Face-to-face interviews using structured questionnaires and standardized qualitative guidelines were conducted to gather information. Descriptive and inferential statistics, including logistic regression models, were applied.

**Results:**

Among 609 study respondents, > 90% agreed to participate in an upcoming targeted mass primaquine treatment (TPT) program. Factors contributing to higher odds of acceptability of the program were older age [adjusted odds ratios (aOR): 2.38, 95% confidence intervals (CI) 1.08–8.96], secondary education level (aOR: 3.99, 95% CI 1.12–20.01), having good knowledge of malaria (aOR: 2.12, 95% CI 1.04–4.76), experiencing malaria within the family (aOR: 1.92, 95% CI 1.14–5.13), and believing eliminating malaria from the village is possible (aOR: 2.83, 95% CI 1.07–4.07). Furthermore, 50 community respondents, 6 midwives, and 4 public health staff (grade II) participated in the qualitative component of the study. Many thought that TPT seemed feasible and stressed that high coverage of underserved groups and health education are needed before commencing the activity.

**Conclusions:**

Most respondents agreed to participate in the proposed mass treatment campaign. Older people with secondary education level and those who had experienced malaria within their families were most likely to report willingness to participate. These same individuals may be important in the community engagement process to increase community acceptance of the program.

**Graphical abstract:**

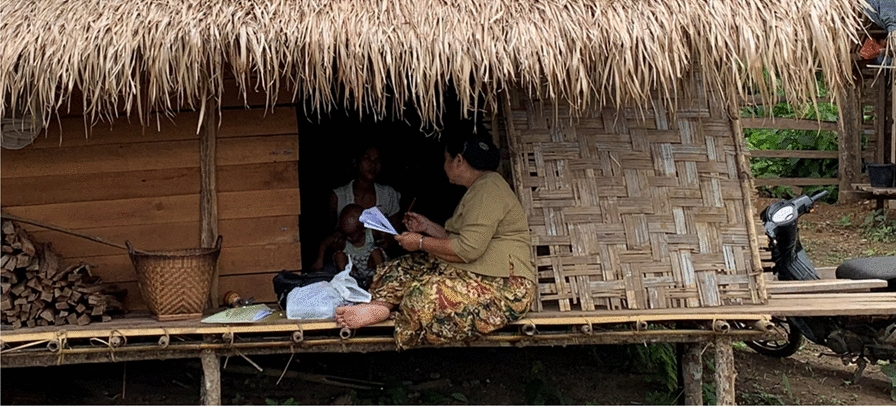

**Supplementary Information:**

The online version contains supplementary material available at 10.1186/s13071-021-05064-y.

## Background

The global malaria burden has been declining, although recently it appears to have stalled. The World Health Organization (WHO) reported an estimated 229 million malaria cases and 0.4 million deaths during 2019 [1]. *Plasmodium falciparum* remains the dominant parasite species globally, but in the Southeast Asia region, *P. vivax* is increasingly the most prevalent malaria species [1, 2]. Nations with both *P. falciparum* and *P. vivax* malaria that are trying to eliminate malaria tend to find a persistent *P. vivax* burden as *P. falciparum* cases decrease. At least one study suggests that most *P. vivax* cases (3 in 4 patients) result from relapses rather than new infections [3]. Relapse from dormant hypnozoites is a challenge to achieving the desired elimination goal [4, 5]. However, no diagnostic test for detecting liver-stage parasites is available [6].

In Myanmar, the malaria problem has been reduced by collaborative efforts between government and non-government organizations, especially since 2011 [2]. There is a nationwide goal of malaria elimination by the year 2030. Yet, 56,640 confirmed cases together with 14 deaths were reported in 2019 [1]. Among the total malaria incidence, 59.0% were from *P. vivax*, a 28% increase in the proportion from 31.0% in 2011 [1, 2, 7]. The provision of easy and socio-culturally appropriate access to diagnosis and treatment facilities has led to a reduction in the incidence of *P. falciparum* [8]. Compared to *P. vivax*, treatment of *P. falciparum* with artemisinin-based combination therapies requires a shorter treatment regimen and has minimal side effects or complications [4, 9].

In 2015, the National Malaria Treatment Guidelines (NMTG) were updated and endorsed by the Department of Public Health, Ministry of Health, Myanmar. It presented the standard treatments for uncomplicated malaria cases to every healthcare provider [10]. Chloroquine (25 mg/kg body weight) followed by 0.25 mg/kg of primaquine (PQ) for 14 days is recommended for the treatment of uncomplicated *P. vivax* infections [10]. Full compliance with the treatment is necessary for achieving radical cure by clearing both blood- and liver-stage *P. vivax* parasites [11–13]. However, poor compliance among *P. vivax* patients and relatively high numbers of patients lost to follow-up regarding completing the full PQ course were reported [14]. The underlying reasons for not completing the 14-day course included complacency among patients after resolution of the symptoms, which often occurs before completing the full course, and simply forgetting to take the medication or carry the medication to the workplace [14]. In a limited resource setting, the directly observed treatment (DOT) strategy for all malaria cases appears impractical unless the incidence is low [7].

Several previous studies have suggested that mass drug administration (MDA) or targeted MDA is feasible and effective for quickly reducing malaria case loads or eliminating the malaria reservoirs [15–17]. Notably, MDA of PQ under close observation effectively reduced relapse cases of *P. vivax* [18]. High population coverage and community participation are essential for the success of this type of public health intervention [18, 19].

A targeted PQ mass treatment (TPT) program has been proposed and approved by the Ministry of Health to first test the safety and feasibility of targeted *P. vivax* elimination in Myanmar. The intervention includes mass administration of PQ targeted specifically to people without glucose-6-phosphate dehydrogenase deficiency and who are living in high *P. vivax* burden villages. Given the importance of having high population coverage, this mixed-methods study was designed to assess the relative acceptability of TPT among study villages in a northern Myanmar township, Banmauk. An underlying goal was to assess the reasons for or against likely participation in TPT among rural villagers in a malaria-endemic setting. The results of this study will be used to inform the implementation of TPT first in a limited study setting, and, if the results indicate that the intervention is safe and effective, it will then be scaled up as a general public health tool for malaria elimination.

## Methods

### Study site

Banmauk Township is located in the Sagaing Region of northern Myanmar and was selected as a study area because of its persistently high malaria burden. In Banmauk, the malaria burden has been declining, with an annual parasite incidence (API per 1000 population) of 11.0, 6.5, and 3.8 in 2016, 2017, and 2018, respectively. *Plasmodium falciparum* accounted for > 80.0% of total infections, but *P. vivax* has become the dominant parasite in some villages. As of 2019, four major organizations (National Malaria Control Program, University Research Co., Population Services International, International Organization for Migration) were intensively implementing malaria control activities in the township, including but not limited to malaria surveillance activities through trained village health volunteers, regular active case detection, awareness-raising campaigns, and distribution of bed nets. The township is gradually moving toward the pre-elimination phase, currently focusing on residual malaria cases and potential transmission foci.

The top five *P. vivax* prevalent villages were selected for this study, with a total population of approximately 3000 people living in 609 households. In each village, at least a government health center or a village health volunteer oversees the village's health aspects. Within a 10-km range, there are no gold mines or other large-income opportunities that might attract migrants or mobile populations to stay at the selected villages. Conversely, this lack of occupational opportunity may lead to the relocation of some villagers away from the study villages. Mass distribution of long-lasting insecticide-treated nets (LLINs) occurred every 3 years, followed by yearly topping-up for high-risk individuals such as pregnant women, small children, and forest-goers. A mixed-methods study was conducted in these five villages where the TPT activity was proposed.

### Study samples

A mixed-methods study design was chosen to combine quantitative approaches, which would represent the five study villages, with a qualitative component, which was meant to provide a more in-depth understanding of the same study villages. For the quantitative study, household leaders or their representative from the five villages were targeted for inclusion. If the household leader could not participate, the next in line from the same household was asked to participate. One person from each of the 609 households was recruited. The quantitative study inclusion criteria included being at least 18 years of age, living in the respective village for at least a year, and being sufficiently healthy to answer questions during an interview.

The qualitative component of this study targeted two groups who would play important roles in the planned TPT campaign: community members living in the selected villages and government healthcare officials working in the selected villages. Ten community members from each village, 50 respondents in total, were recruited for this study using purposive sampling. This sample size was chosen as a balance between a representative sample from the villages and labor and time constraints inherent to qualitative data collection [20]. Inclusion criteria among community members included being > 18 years old, living in the respective village for at least 5 years, and having sufficient knowledge of the overall situation of the villages. Recommendations from village administrators were also considered for participant selection. Furthermore, the sex ratio of participants (aiming for balance), age distribution, and a mixture of different occupations and education groups were also considered. All government healthcare officers assigned to the five selected villages were invited to participate in the qualitative study. Inclusion criteria included having at least 2 years of service. Six midwives and four public health staff (grade II) participated in the study.

### Study tools

A cross-sectional mixed-methods study was deployed in July 2019, which was coincident with the rainy season. For the quantitative part, the face-to-face interviews using structured questionnaires were given to 609 household leaders or their representatives. The questionnaire was first developed in English, based on qualitative data gathered in Thailand and Myanmar. It was then translated into the Myanmar language by the research team. The questionnaire included four main themes addressing: (i) general characteristics of the respondents, (ii) malaria knowledge regarding transmission, diagnosis, symptoms, treatment, and prevention of malaria, (iii) attitudes towards severity, beliefs, and misconceptions, and (iv) practices, including potential agreement to participate in a TPT campaign (Additional file 1).

A standardized qualitative guideline in the Myanmar language was used to collect qualitative data through in-depth interviews (Additional file 1). The first part of the guideline was for reporting general characteristics of the respondents, including age, gender, occupation, and education for community members. In addition, it included the position and experience of healthcare providers. The second part of the guideline was intended for qualitative data collection. The contents for the community addressed previous experiences with malaria and their potential agreement to participate in upcoming TPT exercises. In the guidelines for healthcare provider respondents, there were open questions regarding current malaria trends, practices in prescribing PQ, and possible challenges to implementing TPT.

### Data collection

Four data collection assistants with university degrees were trained for 2 days. The training modules included sessions on orientation to both components (quantitative and qualitative) of the study, objectives, interview procedures, and ethical precautions. Practical sessions were also organized and supervised by PLA, MTS, and MPK. For the quantitative study, the assistants went to the selected villages and contacted respective village leaders to ask for the household listing, including the names of household leaders. Individual households were visited, and face-to-face interviews were conducted. A standardized information sheet was used to explain the nature of the study, flow of the interview, and right to withdrawal from the interview at any time for all potential respondents. An interview took no more than 15 min. Similarly, the face-to-face in-depth interviews for the qualitative component of this study were conducted at each household of respondents. While one data assistant asked the respondent questions, another assistant wrote down the verbatim report. The answers were also recorded. Each in-depth interview took approximately 30 min. The consent forms were signed by the respondents before commencing the interviews. Respondents who were illiterate were asked to provide consent by providing a right thumb print (illustrated on the consent forms). The study team also prevented other persons who could influence the participants' answers from entering the interviewing environment.

### Data entry and analysis

The quantitative data were entered into Microsoft Excel 2018 (Excel for Mac, version 16.16.27, Seattle, WA, USA) spreadsheets and analyzed using the Statistical Package for the Social Sciences (IBM SPSS Statistics for Macintosh, version 23, IBM Corp., Armonk, NY, USA). The data were entered and cross-checked daily to eliminate data entry errors. Descriptive statistics including percentage, mean, median, standard deviation, and minimum and maximum values were presented. In addition, simple logistic and multivariable logistic regression models were used to investigate possible associations between variables and the reported agreement to participate in TPT ("1" if agreed to participate and "0" if not). Crude odds ratios (cORs) and model-adjusted odds ratios (aORs) were calculated together with 95% confidence intervals (CIs).

Sixty in-depth interviews, including the answer sheets from 50 community respondents and 10 healthcare professionals, were also systematically analyzed. All the interview notes were first checked by the lead authors for typos, textual errors, and repetitions. The general characteristics of the respondents were entered into Microsoft Excel 2018 spreadsheets, and cumulated numbers along with the proportions were calculated. Second, the qualitative answers in the Myanmar language were translated into English independently by PLA and MTS. The correctness and completeness of transcripts were thoroughly checked by MPK, LC, and DMP. The intended meanings of the original texts were also ensured by comparing them with English translations. Then, each transcript was coded line by line and roughly grouped under similar topics. The mindJet MindManager software system (version 12) [21] was employed to analyze the textual data and intensively visualize all of the contents for community respondents and healthcare providers. The constructed sentences were finalized and confirmed among all authors until all the conclusions were mutually agreed upon.

## Results

### Quantitative results

#### General characteristics of the quantitative study respondents

More than 60% of respondents were female. The mean age was 41.5 (range 18–85) years. About 47% had attained primary education, followed by 25% with secondary education. Most (88%) worked as farmers. Around 74% were married, and approximately 47% had 6–9 family members. Family sizes ranged from 1 to 13 members, with a median of 6 (Table 1).

**Table 1:** General characteristics of the 609 participants

Characteristics	*n* (%)
Sex	
Male	236 (39)
Female	373 (61)
Age (years)	
18–30	187 (31)
31–40	137 (23)
41–50	112 (18)
51–60	87 (14)
> 60	86 (14)
Mean age ± SD: 41.5 ± 15.5, median: 38, minimum: 18, maximum: 85 years	
Educational level	
Illiterate	102 (17)
Monastery education	62 (10)
Primary education (grade 1 to 9)	284 (47)
Secondary education (grade 10 to 11)	154 (25)
University and above	7 (1)
Occupation	
Unemployed	59 (10)
Farmers	539 (88)
Merchants	4 (1)
General laborers	7 (1)
Marital status	
Single	75 (12)
Married	452 (74)
Others (widowed/divorced/separated)	82 (14)
Numbers of family members	
1–3	61 (10)
4–5	241 (40)
6–9	283 (47)
≥ 10	24 (3)
Mean ± SD: 5.9 ± 2.1, median: 6, minimum: 1, maximum: 13	

SD, standard deviation

#### Knowledge of malaria

Approximately 73% of the participants described malaria as a disease transmitted through an infectious mosquito bite. About 93% reported that the risk of malaria was greater for individuals living in the forest. At least 94% reported that drinking stagnant water was the source of malaria infection (a common misconception in the region) [22, 23]. Most respondents (92%) reported seeking malaria diagnosis services by visiting official healthcare providers. More than 70% mentioned having a blood test for the disease diagnosis. More than 98% of the survey respondents asked other community members who had previously had malaria for advice about their malaria diagnosis and illness.

Approximately 73% correctly described fever with chills and rigor as the cardinal malaria symptoms. Around 30% mentioned headache as one of the symptoms of malaria. Almost 20% answered that cough is a symptom of malaria. When experiencing malaria symptoms, 83% reported that they would seek malaria medication from a malaria post or clinic. Finally, 15% reported that it was possible to be reinfected with malaria.

The last section of the malaria knowledge part addressed malaria prevention. Around 32% knew that avoiding mosquito bites could prevent malaria infection. More than 74% mentioned sleeping under bed nets. Almost 96% reported that not drinking stagnant water could prevent malaria infection. Meanwhile, 98% reported using personal protective measures could prevent malaria infection (Table 2).

**Table 2:** Malaria knowledge among 609 participants

Items	Yes *n* (%)	No *n* (%)
How is malaria transmitted?		
Bite of infectious mosquito	445 (73)	164 (27)
Living in the forest^a^	568 (93)	41 (7)
Drinking stagnant water^a^	572 (94)	37 (6)
How is malaria diagnosed?		
Visiting healthcare professionals	563 (92)	46 (8)
Having a blood test	435 (71)	174 (29)
Asking community members who have had malaria^a^	601 (99)	8 (1)
What are common malaria symptoms?		
Fever with chills and rigor	445 (73)	164 (27
Cough^a^	117 (19)	492 (81)
Headache	184 (30)	425 (70)
How have you sought treatment for malaria?		
Taking herbal medicine^a^	33 (5)	576 (95)
Receiving medicines from malaria clinic/ post	503 (83)	106 (17)
If a malaria patient is cured, can he/she get a malaria infection again?	92 (15)	517 (85)
What do you do to protect yourself from malaria?		
Prevent mosquito bites (other than bed nets)	194 (32)	415 (68)
Sleeping under bed nets	452 (74)	157 (26)
Avoid drinking stagnant water^a^	584 (96)	25 (4)
Using personal protective measures (other than bed nets and other mosquito avoidance measures)	598 (98)	11 (2)

^a^Incorrect response

#### Attitudes about malaria

Most (88%) participants reported that malaria could cause death. Around 71% of them believed that local people could die from malaria. More than 80% of the respondents accepted that every person had an equal risk of infection, and 88% said that malaria infection did not depend on people's economic status. Almost 80% were aware that malaria was a common health problem in their community, and 80% also perceived that malaria infections in children tend to be more severe and/or have adverse consequences. Approximately 72% did not believe that drinking alcohol would relieve malaria. However, 58% were afraid to take antimalarial drugs together with some foods. About 50% of the respondents did not believe that free treatment led to malaria patients not completing the prescribed drugs. More than 50% reported that antimalarial medicines were safe with only a few side effects or complications. For malaria prevention, about 60% reported that house spraying could be effective in preventing malaria. However, 48% thought that health officers were solely responsible for malaria prevention (Table 3).

**Table 3:** Study participants' (*n* = 609) attitudes toward malaria

Statements	Agree *n* (%)	Not sure *n* (%)	Disagree *n* (%)
Malaria can cause death	533 (88)	45 (7)	31 (5)
Local people do not die from malaria^a^	106 (17)	70 (12)	433 (71)
Everyone can acquire malaria	495 (81)	47 (8)	67 (11)
Malaria infection depends on peoples’ economic status^a^	47 (8)	24 (4)	538 (88)
Children have more severe malaria than adults	488 (80)	49 (8)	72 (12)
Drinking alcohol can relieve or cure malaria^a^	47 (8)	124 (20)	438 (72)
People will not complete malaria treatment if it is free^a^	93 (15)	215 (36)	301 (49)
To be completely cured, a patient has to take the full treatment	565 (93)	34 (5)	10 (2)
Antimalarial medicines may have side effects	102 (17)	176 (29)	331 (54)
If you take antimalarials with some foods (e.g. durian) it can make you ill^a^	351 (58)	159 (26)	99 (16)
Malaria prevention is only the duty of health officers^a^	294 (48)	24 (4)	291 (48)
House spraying can prevent malaria	365 (60)	185 (30)	59 (10)
Malaria is a community health problem	482 (79)	70 (12)	57 (10)

^a^Incorrect responses

#### Overall knowledge and attitude levels

Most of the respondents attained a score of 9 (out of 16) for the knowledge part of the survey, with a minimum score of 4 and a maximum of 13. Individuals were categorized as having “good” or “poor” knowledge based on the mean scores for all responses. Approximately half of the respondents were categorized as having good knowledge and good attitude scores. Most respondents achieved 30.4 out of 39 total attitude scores. No one received a full score, and the range was 19–38 (Table 4).

**Table 4:** Overall malaria knowledge and attitude levels among 609 participants

Items	*n* (%)
Knowledge on malaria^a^	
Good	322 (47)
Poor	287 (53)
Mean score ± SD: 9.26 ± 1.9, minimum score: 4, maximum score: 13	
Overall attitude^a^	
Positive attitude	316 (52)
Negative attitude	293 (48)
Mean score ± SD: 30.4 ± 2.9, minimum score: 19, maximum score: 38	

^a^Grouping by mean scores: good: > mean, poor: ≤ mean; SD: standard deviation

#### Malaria diagnosis and treatment and acceptability of targeted mass treatment

As the study site was a malaria-endemic area, 34% of respondents had experience with malaria infections within their families. Among those who had had malaria, 47%, 45%, and 8% sought treatment at one of the malaria clinics, hospitals, and other places such as informal providers, traditional healers, or quacks, respectively. Moreover, 91% of the malaria patients completed treatment. Moreover, 74% reported that eliminating malaria in their community within a reasonable time frame was possible. Among them, 96% recommended including active involvement of healthcare professionals and villagers during elimination efforts. Finally, 95% of the respondents agreed to take part when the TPT program was implemented (Table 5).

**Table 5:** Malaria prevention and treatment practice (*n* = 609)

Items	*n* (%)
Malaria experienced and treatment adherence	
Have any of your family members (including yourself) had malaria?	
No	402 (66)
Yes	207 (34)
If yes, where did the person receive treatment? (*n* = 207)	
Malaria clinic or post	97 (47)
Hospital	94 (45)
Other places (informal providers, traditional healers, or quacks)	16 (8)
Treatment compliance among malaria patients (*n* = 207)	
Took medicine only until symptoms disappeared	18 (9)
Completed treatment	189 (91)
Involvement in future malaria elimination activities	
Can malaria be eliminated from the village?	
Yes	448 (74)
No	85 (14)
Not sure	76 (12)
If yes, who should participate in the elimination activities? (*n* = 448)	
Healthcare professionals	15 (3)
Villagers	2 (1)
Both healthcare professionals and villagers	427 (96)
Acceptability of targeted mass primaquine treatment	
There is medicine that can help eliminate malaria (primaquine), but everyone has to take it continuously for 14 days. Would you take it?	
Yes	576 (95)
No	33 (5)

#### Predictors of reported acceptability of targeted mass primaquine treatment

The associations among general characteristics, overall knowledge and attitudes categories, and acceptability of proposed TPT activity are reported in Table 6. Respondents in the older age groups were more likely to report being willing to participate in TPT. Respondents aged 51 to 60 years (cOR: 1.58, 95% CI 1.06–5.42), > 60 years old (cOR: 2.37, 95% CI 1.07–8.0), and with good knowledge levels (cOR: 2.02, 95% CI 1.60–2.43) and respondents who think malaria can be eliminated from the villages (cOR: 2.24, 95% CI 1.06–2.91) all had higher odds of being willing to participate in TPT. In the multivariable logistic regression, those who had attained secondary education (aOR: 3.99, 95% CI 1.12–20.01) and those who had experienced malaria within the family (aOR: 1.92, 95% CI 1.14–5.13) had greater odds of being willing to participate in TPT.

**Table 6:** Associations among general characteristics, knowledge, attitudes and practices, and acceptability of targeted mass drug treatment (*n* = 609)

Characteristics	Not accepted TPT	Accepted TPT	cOR, 95% CI	aOR, 95% CI
	*n* (%)	*n* (%)		
Sex				
Male	13 (2)	223 (37)	Comparator	
Female	20 (3)	353 (58)	1.03 (0.51–2.11)	0.64 (0.27–1.86)
Age (years)				
18–30	7 (1)	180 (30)	Comparator	
31–40	5 (1)	132 (22)	0.80 (0.23–2.8)	0.65 (0.11–3.89)
41–50	8 (1)	104 (17)	0.78 (0.20–2.98)	0.69 (0.12–4.12)
51–60	9 (2)	78 (13)	1.58 (1.06–5.42)	1.47 (1.02–6.72)
> 60	4 (1)	82 (14)	2.37 (1.07–8.0)	2.38 (1.08–8.96)
Educational level				
Primary education	8 (1)	268 (44)	Comparator	
Illiterate	3 (1)	99 (16)	1.02 (0.09–11.05)	0.90 (0.05–10.99)
Monastery education	6 (1)	56 (9)	3.59 (0.51–25.09)	2.56 (0.01–14.66)
Secondary education	16 (3)	146 (24)	3.67 (0.78–17.23)	3.99 (1.12–20.01)
Occupation				
Farmers	25 (5)	423 (70)	Comparator	
Unemployed	8 (1)	51 (8)	2.65 (0.59–11.92)	1.36 (0.44–9.90)
Others (merchants and general laborers)	2 (1)	9 (2)	3.76 (0.23–62.35)	3.55 (0.19–45.28)
Marital status				
Married	29 (5)	423 (70)	Comparator	
Single	1 (1)	74 (11)	0.20 (0.01–6.93)	0.11 (0.01–6.61)
Others	3 (1)	79 (12)	0.55 (0.06–4.75)	0.56 (0.06–4.88)
Number of family members				
1–3	4 (1)	57 (10)	Comparator	
4–5	11 (2)	230 (37)	0.77 (0.13–4.52)	0.87 (0.11–4.63)
6–9	16 (2)	267 (44)	0.53 (0.11–2.53)	0.63 (0.11–3.29)
≥ 10	2 (1)	22 (3)	0.66 (0.14–3.03)	0.72 (0.13–4.05)
Knowledge level				
Good	16 (3)	306 (50)	2.02 (1.60–2.43)	2.12 (1.04–4.76)
Poor	17 (3)	270 (44)	Comparator	
Attitude level				
Positive attitude	17 (3)	299 (49)	1.02 (0.50–2.05)	0.89 (0.41–1.92)
Negative attitude	16 (3)	277 (45)	Comparator	
Have any family members including yourself had malaria?				
No	21 (3)	381 (63)	Comparator	
Yes	12 (2)	195 (32)	0.90 (0.43–3.86)	1.92 (1.14–5.13)
Can malaria be eliminated from the village at some point?				
No	3 (1)	82 (14)	Comparator	
Not sure	10 (1)	66 (11)	0.31 (0.14–0.69)	0.24 (0.09–0.61)
Yes	20 (3)	428 (70)	2.24 (1.06–2.91)	2.83 (1.07–4.07)

95% CI, 95% confidence interval; cOR, crude odds ratio by simple logistic regression; aOR, adjusted odds ratio by multiple logistic regression

Of note, seven participants with higher education levels and expressing acceptance of the treatment were excluded from the analysis

### Qualitative results among villagers

A total of 50 villagers participated in the qualitative interviews. Around two-thirds were 31–60 years old, and 62% were male. Most (88%) worked as farmers, followed by a few of mixed employment types, including village leaders, general laborers, and merchants. More than half (54%) had a primary-level education. Six midwives and four public health workers with > 2 years of working experience joined as the study respondents.

#### Experience with malaria

There was still a considerable prevalence of malaria-like symptoms (fever with chills and rigor), and most of the respondents had contracted malaria infections. The respondents described harsh malaria situations within their communities in previous years, including deaths and other complications from malaria infection:

“…malaria was a very fatal disease in the old days…some patients could not catch up the time for life-saving even if they went to the township level for advanced treatment.” (IDI, villager)

Some respondents who had contracted malaria had ceased taking their medication after symptom relief. Many could not correctly describe the full course of prescribed antimalarial medicines, including the total number of days, total number of tablets, and drug-taking schedule.

“…malaria was diagnosed by a blood test at the health center…I had to take the drugs for one time per day for 6 days…I stopped taking the drugs after symptoms disappeared.” (IDI, villager)

#### Willingness to participate in TPT (targeted primaquine mass treatment)

Almost all the respondents agreed to participate in the upcoming mass treatment activity as they hoped it could eliminate malaria from their villages. All persons guaranteed that they would completely take the full course of the given drug and encourage their children to strictly follow the activity. Drug compliance is more likely to be poor among children who are physically healthy and do not have experience with malaria.

“… our family, as well as myself, will certainly be involved in that project together with the local authority and health staff…whereas we have to encourage our children for good compliance.” (IDI, villager)

### Qualitative results among healthcare providers

#### Current malaria situation and experience with PQ prescription

The healthcare respondents overseeing the proposed villages where TPT has been proposed described the serious malaria situation in the past and current improved conditions. The malaria problem has seemingly declined in this area, but there were still many cases, especially in the rainy season (approximately May through October). Meanwhile, higher prevalence was usually observed among villagers who stayed in gold mining areas (away from their home villages) and returned to their residences after a period. The respondents also discussed the possibilities of relapses of *P. vivax* malaria in the study villages. As the malaria incidence was continuously declining, the healthcare respondents believed that malaria transmission could be interrupted after some time.

“…malaria burden has been decreasing especially in this decade…it remained highly prevalent during the rainy season while there were very rare cases in other times…it is common among gold miners who worked at gold mine areas in other villages…there were likely to be some relapsed cases of *P. vivax* malaria infection as well.…the transmission of malaria could be stopped soon.” (IDI, healthcare provider)

All the healthcare provider respondents had extensive experience in treating malaria cases. They could also describe the true purposes of giving PQ in both *P. falciparum* and *P. vivax* infections. The respondents recognized the importance of eradicating latent *P. vivax* hypnozoites by giving prolonged PQ regimens (i.e. for 14 days). However, some *P. vivax* patients had poor compliance with the drug treatment regimen, especially after malaria-like symptoms disappeared. So far, the healthcare respondents have not noticed any adverse complications or severe consequences among the patients after prescribing PQ.

“…we currently use primaquine regimens…single dose in *P. falciparum* malaria for interrupting onward transmission, while a 14-day course in *P. vivax* for eradicating liver-stage parasites…no special issues existed except poor compliance of patients upon prolonged duration of primaquine treatment.” (IDI, healthcare provider)

#### Possible challenges to the implementation of TPT

Some officer respondents believed that TPT was feasible, and they were eager to assist with the project. However, they also noted some possible challenges that might arise during the implementation of TPT activities. Most healthcare professionals advised considering coverage for some hard-to-reach people such as migrant workers who usually live in forest-related workplaces for their incomes. As the drugs have to be taken for 14 consecutive days, the involvement of those workers would be a critical issue.

“…for the success of every intervention, at least there should be a proper collaboration of the community including all the villagers as well as migrant workers who usually are lost to follow-up.” (IDI, healthcare provider)

Many provider respondents also stressed that health education was crucial to ensure the villagers complete the full primaquine regimen. They also suggested possible channels for effective dissemination of health messages based on their experience and geographical features of the study areas.

“… the health messages should be disseminated through formal health-talks, pamphlets… as there is no electricity here, video presentations by mobile phone will be more feasible [than television]…the information should cover advantages after receiving mass treatment, disadvantages of poor compliance, correct prevention methods, and finally care-seeking behaviors when malaria is suspected.” (IDI, healthcare provider)

## Discussion

As part of malaria elimination efforts, Myanmar is considering several innovative, targeted approaches, including MDA with PQ (targeted PQ treatment or TPT). MDA is heavily dependent on community buy-in and participation [24]. This study noted some likely challenges to implementing TPT in the target villages, but > 90% of the survey respondents expressed their acceptance of the upcoming TPT activity. Nevertheless, completing the full drug course and full participation in all follow-up assessments should be ensured by comprehensive community engagement and management of the mass drug administration.

Several respondents’ characteristics were strongly associated with reporting that they would participate in the upcoming TPT. Respondents who were older (≥ 51 years old), had secondary education, had higher than the mean understanding of malaria, had family members who had experienced a malaria infection, and believed that malaria could be eliminated from their village all reported that they would participate in the TPT.

Many of their attributes align well with the Health Belief Model (HBM), a theoretical framework developed to understand health behaviors [25]. The HBM is based on the concept that individual participation in health interventions is based on a balance between perceived risks and benefits along with barriers to participating in the intervention [25–27]. Following this line of thinking, it is perhaps not surprising that respondents who have a greater understanding of malaria, who have experienced malaria themselves, who have lived in times when malaria was a major community health problem, and who believe that the intervention could help eliminate malaria from their village reported being willing the participate. The discussions that occurred in our in-depth interviews also corroborate this.

Importantly, community respondents also mentioned some potential challenges, but given the overwhelming reported acceptability, it appears that they see the potential benefits of participating as outweighing the potential difficulties in TPT. In particular, respondents mentioned that children (who have less experience with malaria) might need to be encouraged to participate in the TPT by their parents.

The healthcare provider respondents also suggested two possible challenges for the TPT campaign. The first challenge they mentioned was achieving good participation in hard-to-reach demographic groups such as migrants. They suggested that having dedicated health staff responsible for migrant workers would help with this particular challenge. The healthcare provider respondents also stressed the need for community engagement and health education prior to organizing the mass treatment program to bolster the community buy-in. Previous research recommended that a simple specific education session with short-point contents should be conducted in this area to quickly and efficiently disseminate the information [28, 29].

## Conclusions

This study revealed a high level of acceptability of the TPT program to eliminate vivax malaria by the village respondents. Respondents who had experienced malaria (themselves or their family or community members) and who believed that malaria can be eliminated from their village were most likely to report willingness to participate. Both villagers and health workers described some likely challenges to the mass administration of PQ, and these challenges will need to be addressed during the implementation to ensure sufficient community participation.

## Supplementary Information

**Additional file 1.** Components of the quantitative questionnaire and qualitative guidelines.

## Data Availability

All the data analyzed for this study are already included within the article.

## Abbreviations

API: Annual parasite incidence
aOR: Adjusted odds ratio
CI: Confidence interval
cOR: Crude odds ratio
DOT: Directly observed treatment
HBM: Health Belief Model
IDI: In-depth interview
LLIN: Long-lasting insecticide-treated nets
NMTG: National Malaria Treatment Guidelines
MDA: Mass drug administration
PQ: Primaquine
SD: Standard deviation
SPSS: Statistical Package for the Social Sciences
TPT: Targeted mass treatment
VBDC: Vector-borne disease control
WHO: World Health Organization
