# Supplementary material for: The acceptability of targeted mass treatment with primaquine for local elimination of vivax malaria in a northern Myanmar township: a mixed-methods study
Source: Parasit Vectors. 2021 Oct 24;14:549. doi: 10.1186/s13071-021-05064-y (PMC8543804; doi:10.1186/s13071-021-05064-y)
Supplement: Supplementary file 1 — Additional file 1. Components of the quantitative questionnaire and qualitative guidelines. [file 13071_2021_5064_MOESM1_ESM.docx]

**Additional file 1**

**1. Parts of the quantitative questionnaire**

(a) General characteristics of the respondents: information regarding sex, age in years, education attainments, types of occupation, marital status and numbers of family members were collected.

(b) Knowledge regarding transmission, diagnosis, symptoms, treatment and prevention of malaria: this section included a total of 16 mini questions with ‘yes’ or ‘no’ choice. Both positive and negative questions were structured. Whenever a participant answered the right answer, ‘1’ score was given while ‘0’ score for a wrong answer. The cumulative scores of ‘16’ could be achieved. The overall score of each participant has been categorized as good or poor knowledge group by applying more than or less than the mean score i.e. >mean: good knowledge, ≤mean: poor knowledge.

(c) Attitude towards severity, historical believes and misconceptions: this portion came up with 13 mini-statements including both positive and negative meanings. The 3-points Likert’s scale model was applied and participants could response either they agreed, not sure or disagreed after careful listening to the reading by the interviewer. For a positive statement, the score was given as ‘3’ for agree, ‘2’ for not sure and ‘1’ for disagree. Reciprocally for a negative statement, ‘1’, ‘2’ and ‘3’scores were provided for agree, not sure and disagree, respectively. Then the total score for each participant was calculated and grouped into positive or negative category based on the mean score as >mean: good attitude and ≤negative attitude. The minimum score would be 13 and the maximum one should not be larger than 39.

(d) Malaria practice: from each respondent, the information on presence of previous experienced of malaria infection within the family was gathered. For the participants who agreed with the practice mentioned in the questionnaire, the detailed behaviors of that practice were also identified. Moreover, individual’s perspective towards their involvement on future malaria elimination activities and the last, their agreements to involve in next targeted mass PQ treatment program have been interviewed.

**2. Guideline for qualitative data collection (Community)**

(a) Have you and your family experienced fever with chills and rigors?

Yes/no: How was it and its outcomes?

(b) Have you and your family ever suffered malaria?

Yes/no: If yes, how did you know it was malaria? How many days did you take medicine?

(c) Are there many people sick of malaria in your village? Do you think that how malaria could affect the socioeconomic status and wellbeing of you and your family?

(d) Do you consider malaria as community health problem?

Yes/no: why?

(e) Do you think malaria can be eliminated?

If yes, how can it be eliminated? Who should participate in the elimination? Would you willing to participate? How about your family?

(f) There is an effective medicine for preventing malaria in the community, but everybody has to take it for 14 days, would you take it?

If yes, would you allow your children take it? Why?

If yes, do you think other people would take it? If no, how can we persuade them?

If no, would you kindly give the reason for not taking this effective medicine?

(g) Have you experienced adverse‐side effects from antimalaria drugs or any drug?

If yes, please discuss as much as you can.

**3. Guideline for qualitative data collection (Healthcare provider)**

(a) How do you consider malaria problem in your responsible area?

Probe: for the reason for malaria considered as community problem or the reason for not considered as a community problem.

(b) Do you think we can eliminate malaria in your area?

If yes, who should be involved and how can we do it.

(c) Can you briefly discuss about Primaquine, mainly on its action, detailed prescriptions and any other special experienced of giving this medicine? Any issues or concerns?

(d) Do you think that whether it is possible to make people take part in the targeted mass primaquine treatment (TPT) for 14 days? Would you also willing to collaborate in this activity?

Probe: reason for yes and no.

(e) What will be the possible challenges to implement TPT activity in your area?

(f) What would be the effective channel for send the health-related messages to the community?

Probe: contents, mechanism, and other things.
